# Analytical kernels for efficient constant Q transforms in dark matter searches with LIGO

**DOI:** 10.1038/s41598-025-33428-2

**Published:** 2026-04-01

**Authors:** Alexandre S. Göttel, Vivien Raymond

**Affiliations:** 1 Gravity Exploration Institute, Cardiff University, Cardiff, CF24 3AA UK; 2 Nottingham Centre of Gravity & School of Mathematical Sciences, University of Nottingham, University Park, NG7 2RD Nottingham, UK

**Keywords:** Mathematics and computing, Physics

## Abstract

We introduce a novel scalable logarithmic spectral estimation method based on insights from computer-music analysis, allowing us to perform an efficient constant Q transform with no need for pre-computing. By leveraging symmetries between the time and frequency domains, this method matches the computational efficiency of FFT-based algorithms without, unlike such algorithms, compromising precision. We apply our algorithm to data from LIGO’s third observing run, using the results to enhance a previous search for scalar dark matter which relied on FFT-based approximations. Our results show that we can simultaneously boost the signal-to-noise ratio up to the theoretical maximum and reduce computational costs. With potential for further refinements, this method is already capable of boosting the scientific potential of any logarithmic spectral analysis with limited computational budget, which includes all future dark matter searches with gravitational-wave detectors.

## Introduction

It is by now well-established that gravitational-wave detectors can not only be used to probe different candidates of ultralight dark matter (ULDM)^1–4^, but often provide the most sensitive measurements. The investigated interactions vary wildly between candidates, ranging from altering physical constants^5^, to modifying the behaviour of laser beams^6,7^, accelerating optical components^8–10^, affecting existing gravitational-wave signals^11^, or even being detectable through gravitational interactions^12–14^. However in practice, all analyses share a common fundamental problem: the velocity distribution of virialized dark matter in our galaxy which, when considering the movement of our solar system w.r.t. dark matter particles, results in a Doppler-broadened linewidth of $\delta _f = f\cdot 10^{-6}$^15^ in observed signals. In the case of ultralight dark matter searches, this intrinsic feature results in coherence times being inversely proportional to the candidate’s Compton-frequency (*i.e.* mass). For reference, the LIGO-Virgo-Kagra interferometers^16–18^ are sensitive in a frequency range from ${{10}\, \hbox {Hz} \,to\, {5000}\, \hbox {Hz}}$, ${{10}\, \hbox {Hz}\, to\, {5000}\, \hbox {Hz}}$, corresponding to coherence times $\tau _c$ between about 1 day and 3 minutes. This orders-of-magnitude difference represents a significant technical challenge common to all interferometer-based dark matter searches, as frequency resolutions adapted to one end of the spectrum will be inappropriate for the other.

A variety of approaches have been used in ULDM searches. For instance, one analysis of LIGO data used coincident half-hour segments to cross-correlate the spectra from both interferometers^8^. Although cross-correlation offers significant advantages, the short and constant integration time—chosen to avoid high computational costs—reduces sensitivity. Specifically, when the FFT duration $T_\text {FFT}$ is shorter than the coherence time $\tau _c$, the lower frequency resolution causes more noise to contaminate the signal bin. Other methods^4,19^ used a combination of FFTs with varying integration times piecewise adapted to the coherence times. This is in practice a good approximation, but of course a balance is struck between precision and the number of FFTs. Finally, a study on GEO600 data^3,20^ used a full “Logarithmic power spectral density” (LPSD, also referred to as “constant-Q transform”) approach, where the coherence time is perfectly adapted to every frequency bin. While this creates, by definition, an optimal spectrum for the searches, it is extremely computationally expensive. This is because the varying window size in every frequency bin makes the use of the FFT algorithm impossible, and thus requires $N^2$ operations instead of $N\log N$, where *N* is the number of data points. On GEO600 data, with a minimum frequency of ${{50}\, \hbox {Hz}}$ (corresponding to $\tau _c \approx {{6}\,\hbox {h},}$), this analysis required well over $10^6$ cpu-hr where an FFT would have required less than ${{1}\,\hbox {h}}$. In LVK detectors, the same analysis down to a minimum frequency of 10 Hz would be about a factor 7 more costly but the increased amount of available data (as used in^4^) increases this number by at least another order of magnitude. In LISA^21^, analyzing 200 h of data in a range from 0.1 mHz to 1000 mHz would be about a factor 12 more costly, but given the coherence time of over 200 years at 0.1 mHz, it is not a straightforward comparison. In light of these numbers, one can see that LPSD cannot be run at scale.

This paper introduces a new method which, for the first time, is able to perform the full LPSD calculation while keeping computational costs low and without the need for approximations or pre-computation. Our approach is based on the work in^22^, where terms of the transform (labelled *kernel*, see below) are identified as being very sparse in the frequency domain. One can then greatly speed-up the analysis through zero-suppression. While this works well in computer-music applications where segments are short, the computation of this *kernel* in LIGO would still be too costly. We instead developed an analytical framework to bypass these calculations completely. This delivers optimal results while being up to an order of magnitude faster than a previously-used FFT-based method^4^. This makes it possible to perform optimal interferometric ULDM searches at scale and can be applied to any candidate or subsequent spectral analysis method. We then show this already provides benefits to current results and will be even more advantageous in future runs and for next-generation instruments. Sec. II introduces the problem of scalar field dark matter searches in more detail, Sec. III describes the new method alongside an FFT-based approach, and Sec. IV compares the results of both.

## Scalar field dark matter in LIGO

As discussed in^4^, scalar field Dark Matter originating in the early universe would behave today as a coherently oscillating classical field^26,27^:

1 $$\begin{aligned} \phi (t,\vec {r}) = \phi _0 \cos \left( \omega _\phi t - \vec {k}_\phi \cdot \vec {r}\right) , \end{aligned}$$

where $\omega _\phi$ is the Compton frequency of DM, and $\vec {k}_\phi$ its wave vector. Assuming that the field is responsible for the local DM density $\rho _\textrm{local}$, the amplitude is given by:

2 $$\begin{aligned} \phi _0 = \sqrt{2\rho _\textrm{local}}/m_\phi , \end{aligned}$$

where $m_\phi$ is the Compton mass of DM. Following^28,29^, we consider linear couplings to the standard model Lagrangian:

3 $$\begin{aligned} \mathcal {L}_{\text {int}} \supset \frac{\phi }{\Lambda _\gamma } \frac{F_{\mu \nu }F^{\mu \nu }}{4} - \frac{\phi }{\Lambda _e} m_e \bar{\psi }_e \psi _e, \end{aligned}$$

where $\psi _e$, $\bar{\psi }_e$ are the Standard Model electron field and its Dirac conjugate, respectively, $F_{\mu \nu }$ is the electromagnetic field tensor, $m_e$ is the electron rest mass, and $\Lambda _\gamma$, $\Lambda _e$ represent the couplings. This does not restrict us to any particular model of ULDM, but rather allows us to investigate the most straightforward Standard Model deviation.

Since LIGO’s calibrated data is provided as *gravitational-wave-induced* strain, but our analysis requires a strain that reflects the interferometer’s response to *DM-induced* oscillations, we follow the procedure outlined in^4^ to map LIGO data to DM coupling constants. This yields:

4 $$\begin{aligned} h(\omega )\cdot A_\textrm{cal}(\omega ) \approx \left( \frac{1}{\Lambda _\gamma } + \frac{1}{\Lambda _e}\right) \cdot \frac{\hbar \,\sqrt{2\,\rho _\textrm{local}}}{m_\phi \, c}, \end{aligned}$$

where $h(\omega )$ is the *gravitational-wave-induced* strain at a frequency $\omega$, and $A_\textrm{cal}$ is a conversion factor containing transfer functions obtained through detailed optical simulations^4^. We note that these simulations automatically include effects stemming from a finite light travel time, as discussed in^30,31^.

Assuming that dark matter is virialized as in a standard galactic halo model, its velocity distribution is expected to follow a Maxwell-Boltzman distribution:

5 $$\begin{aligned} f(\vec {v}) = \sqrt{\frac{2}{\pi }}\frac{|v|^2}{\sigma _v^3}e^{-\frac{1}{2}\frac{|v|^2}{\sigma _v^2}}, \end{aligned}$$

where $\sigma _v = \Theta _0\cdot \sqrt{3/2}$ is the characteristic virial dispersion velocity and $\Theta _0 = (229 \pm 1)\, \hbox {km}\, {\hbox {s}^{-1}}$^32,33^. Practically, we model the DM as isotropic and homogeneous in the galactic halo, with a cutoff at the escape velocity $v_{\text {esc}} = (528 \pm 25) \hbox {km}\, {\hbox {s}^{-1}}$^34^. The observed DM frequency $\omega _{\text {obs}} = \omega _\phi + \frac{1}{2}m_\phi \vec {v}_{\text {obs}}^2$ is expected to have a Doppler-broadened linewidth $\delta _f$. This results in a finite coherence time $\tau _c = (\sigma _v/c)^2/\omega _\phi$ for the DM signal^15^. With the solar system moving at the galactic circular velocity $\Theta _0$ and taking Earth’s orbit into account, we estimate $\delta _f \approx f\cdot 10^{-6}$.

## Methods

ULDM frequency-space searches must adapt integration times to the varying coherence times to avoid losing signal contributions and minimise noise^3^. We achieve this using a Fourier transform, but since $\tau _c$ is frequency-dependent this cannot be performed with the Fast Fourier Transform (FFT) algorithm. This represents a significant technical challenge, because a Fourier Transform will require $N^2$ operations instead of the $N\log _2 N$ required by an FFT, resulting in a factor ${\mathcal {O}}(10^{8})$ slowdown at our lowest explored frequencies (${10 \, \hbox {Hz}}$, corresponding to $\tau _c \approx {28}\, \hbox {h}$). This section introduces methods to perform this calculation without having to perform all “brute-force” operations.

We hereinafter use Logarithmic Power Spectral Density (LPSD) nomenclature from^35^. As such, we can re-write the equation for a discrete Fourier transform (DFT) as:

6 $$\begin{aligned} X_j = \sum _{n=0}^{N_j-1} x_n w_{n,j} e^{-2\pi in\mathcal {Q}/N_j}, \end{aligned}$$

where $X_j$ is the Fourier coefficient in the *j*-th frequency bin, the index *n* sums over time, *x* represents our data, $w_{n,j}$ is a window function, and $N_j$ is the frequency-dependent number of data points associated with the coherence time $\tau _c$. We used:

7 $$\begin{aligned} \mathcal {Q} = f / \delta _f. \end{aligned}$$

With $\mathcal {Q}$ being constant, the entire frequency dependence of the exponential term is determined by $N_j$, again highlighting the impossibility of applying a standard FFT. Further, assuming that the frequency axis is made of *J* logarithmically-spaced bins between the analysis’ minimum and maximum frequencies f$_{min}$ and f$_{max}$, the frequency $f_j$ in each bin *j* is given by:

8 $$\begin{aligned}&\text {f}_j = \text {f}_{min}e^{\frac{j}{J - 1}(\log \text {f}_{max} - \log \text {f}_{min})}. \end{aligned}$$

Since, by definition, the frequency spacing $\text {f}_{j+1} - \text {f}_j = \text {f}_j / \mathcal {Q}$, we further arrive at:

9 $$\begin{aligned} J = 1 + \frac{\log \text {f}_{max} - \log \text {f}_{min}}{\log (1 + \mathcal {Q}) - \log (\mathcal {Q})}. \end{aligned}$$

Unsurprisingly, *J* depends only on the width of the frequency axis and our resolution term $\mathcal {Q}$. Finally, the discrete integration length $N_j$ (corresponding to our coherence time) can simply be defined as $\text {f}_s / (\text {f}_{j+1} - \text {f}_j)$, which, using Eqs. (8) and (9), leads to:

10 $$\begin{aligned} N_j&= \frac{\text {f}_s}{\text {f}_{min}}\mathcal {Q}\cdot \left( \frac{\mathcal {Q}}{1 + \mathcal {Q}}\right) ^j, \end{aligned}$$

where f$_s$ is the sampling frequency.

To calculate the power spectrum, we follow an approach based on the Welch method^36^ and used in^4,35^, which summarizes to:

11 $$\begin{aligned} A_j^{total} = \frac{2}{f_s}\frac{\frac{1}{S_j}\sum _{s=0}^{S_j-1} |X_{j, s}|^2}{\sum _{n=0}^{N_j-1} w_{n,j}^2}, \end{aligned}$$

where $A_j^{total}$ is the power spectral density in the frequency bin *j*, the power $X_j$ is averaged over data sections, labelled with the subscript *s*, $S_j$ is the number of data sections for the Welch average, and $w_n$ is a window function. As one can see, the term in the numerator is simply an average of the power, while the denominator is a frequency-dependent normalisation term. We use an overlap of 0.2 for the Welch averaging, again for consistency with previous works^3,4^. To help visualize the scale of the analysis, a list of commonly used variables and their values in this study can be found in Table 1.

### FFT approximation

As seen above, the dependence of $N_j$ on frequency prohibits the use of a simple FFT. However, it is also clear from Eq. (10) that the frequency-dependence is quite slow ($\propto (1 + \mathcal {Q}^{-1})^j$, where $\mathcal {Q} \gg 1$). A natural solution to those computational problems is thus to make approximations related to $N_j$. This idea was used before, *e.g.*^19,37^ where the data was split in 10 Hz-wide frequency bands for FFTs, and in^4^ we instead defined the length of each frequency band by requiring that the deviation from the optimal coherence time remains within a certain fraction $\epsilon$. Specifically, using Eq. (10) we set:

12 $$\begin{aligned}&N(j+\delta j) \le (1 - \epsilon ) N_j\end{aligned}$$

13 $$\begin{aligned} \leftrightarrow \ &\delta _j^\text {max} = \frac{\log (1 - \epsilon )}{\log (\mathcal {Q}) - \log (1 + \mathcal {Q})}. \end{aligned}$$

With LIGO, $\delta _j^\text {max} \approx 10^4$ when $\epsilon = 1\%$. This very slow variation of $N_j$ over frequency thus allows us to simply set *N* to be constant over frequency bands of (up to) $\delta _j^\text {max}$ bins. Defining these blocks in terms $\delta _j^\text {max}$, as opposed to a specific width in Hz, ensures that the deviation from the optimum never exceed the threshold set by $\epsilon$, which is equivalent to a threshold in SNR loss.

**Fig. 1:**
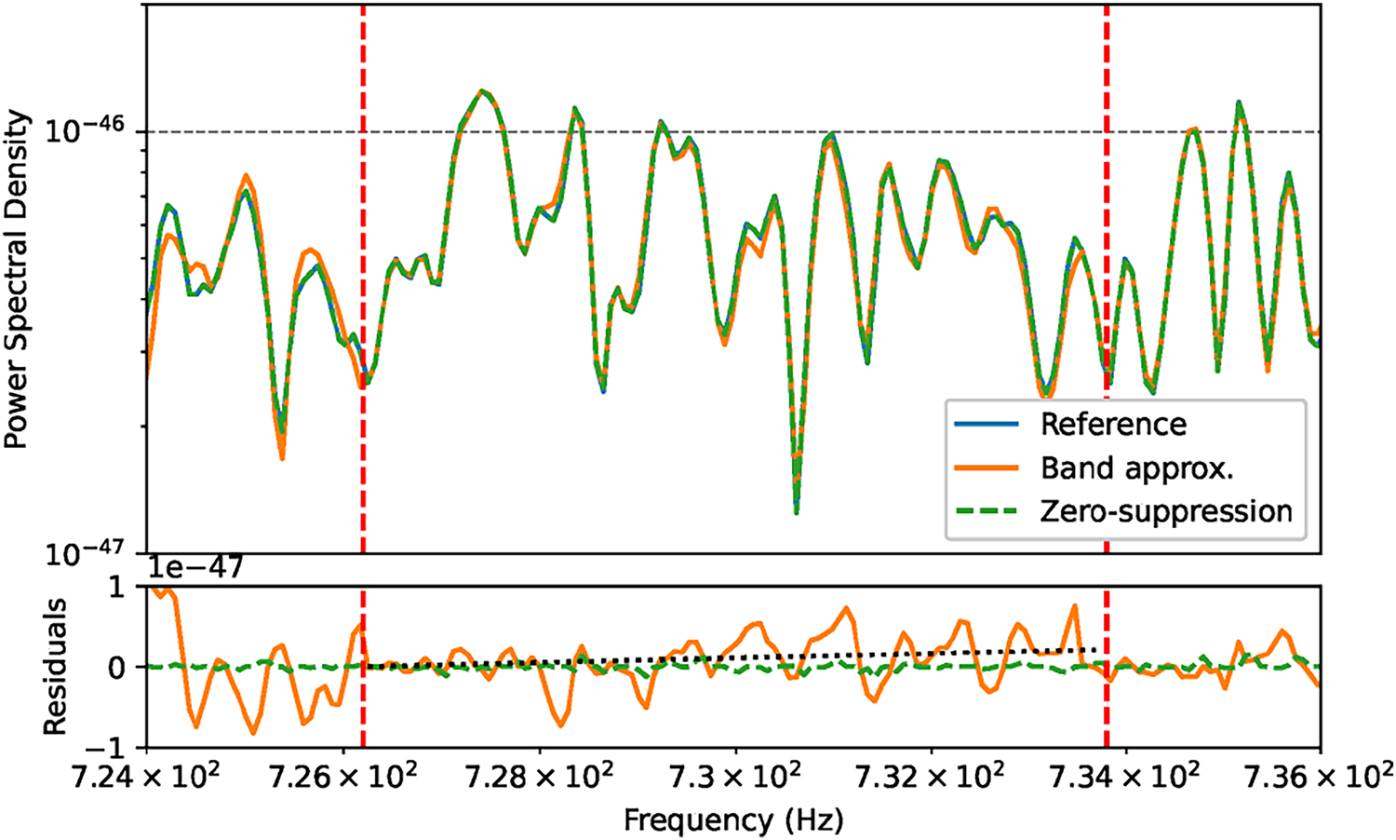
PSD and residuals between methods: the solid orange lines depict FFT approximation results ($\epsilon = 1\%$), the dashed lines the zero-suppression results. The reference solid blue line (LPSD) from a brute-force calculation is fully obscured by the dashed line, showing, as reflected in the residuals, how precise the zero-suppression is. A full band, roughly between 726 Hz to 733 Hz, is marked by vertical lines. A gradual *drift* is exemplified by the dotted line, resulting from a least-squares fit to the residuals from the FFT approximation results.

As demonstrated in^4^, this method can be used competitively while providing a $\mathcal {O}(10^4)$ speed-up. However, it does have a few drawbacks, one of them naturally being the sub-optimal integration time - though the overall SNR loss is small^3^. However, as seen in Fig. 1, the structure also induces a *drift* in frequency as the sub-optimal $N_j$ diverts from its corresponding $\tau _c$, which must be corrected for in an additional post-processing step.

### Spectral zero-suppression

**Fig. 2:**
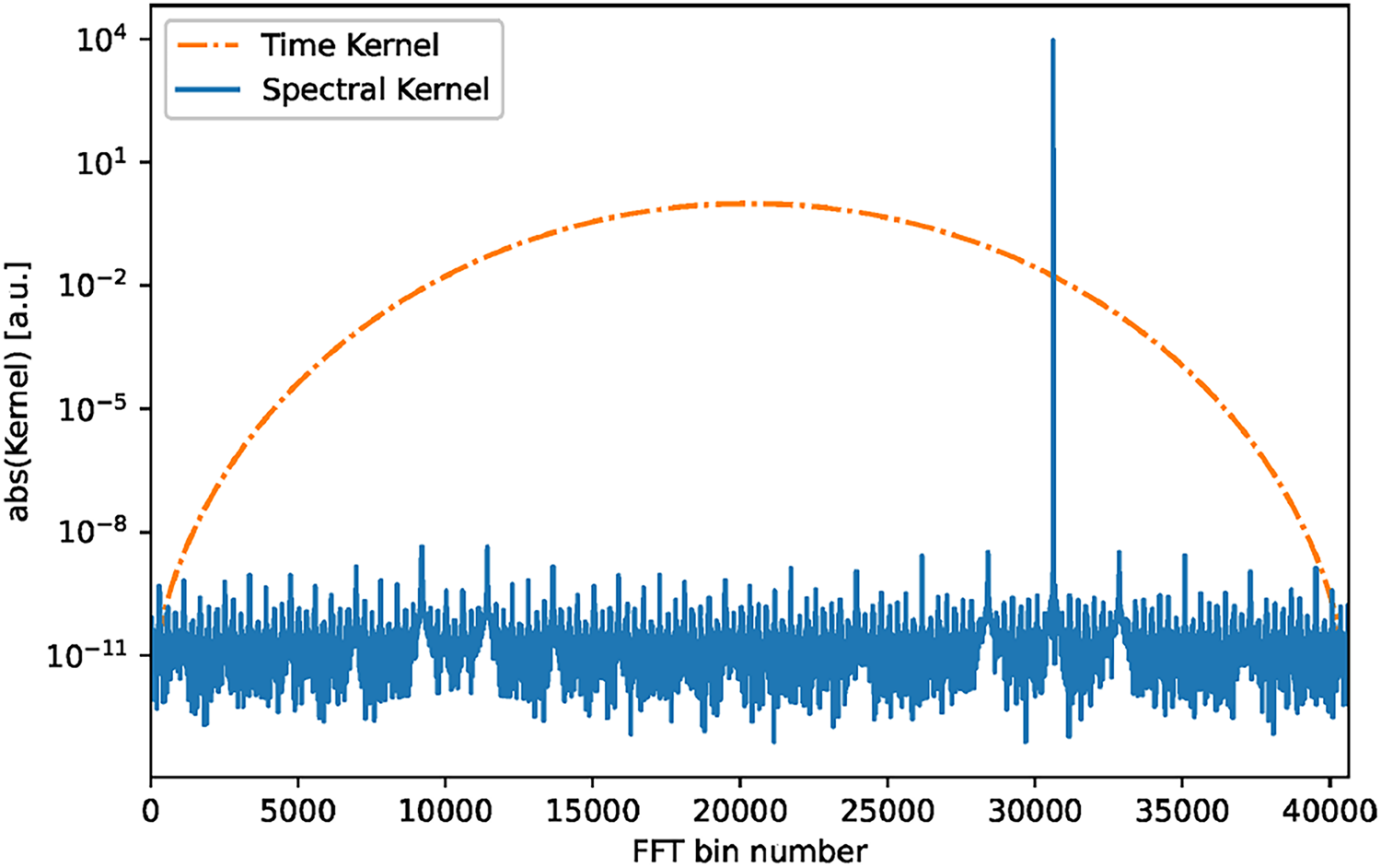
Absolute value of a time kernel (dot-dashed) and its associated spectral kernel (solid) for an example frequency. As one can see, the spectral kernel is much more sharply peaked, to the point where most values (scattered around $10^{-11}$) are dominated by float precision errors.

This section introduces an approach based on that developed originally for computer-music applications in^22^. The basic idea is to identify the terms in Eq. (6) with a data term *x* and a *kernel* $\mathcal {K}$:

14 $$\begin{aligned} X_j = \sum _{n=0}^{N_j-1} x_n w_{n,j} e^{-2\pi in\mathcal {Q}/N_j} = \sum _{n=0}^{N_j-1} x_n\mathcal {K}^*_{n,j}, \end{aligned}$$

where the asterisk denotes the complex conjugate, in order to make use of Parseval’s theorem:

15 $$\begin{aligned} \sum _{n=0}^{N-1} x_n\mathcal {K}^*_n = \frac{1}{N}\sum _{k=0}^{N-1}\tilde{X}_k\tilde{K}_k^*, \end{aligned}$$

where $\tilde{X}$ and $\tilde{K}$ represent the Fourier transforms of *x* and $\mathcal {K}$, respectively. The reason for using this identity becomes apparent in Fig. 2, where one can see that while the kernel is very broad in the time domain, it is very sharply peaked in the frequency domain. Indeed, in the context of a LIGO dark matter search, the lowest analysed frequency (10 Hz) is associated with a DFT length of $N(0) \approx 1.7\cdot 10^9$, but the number of non-zero bins in the spectral kernel is about ten (where we define zero-bins as those contributing negligibly to the sum in Eq. (15)). Therefore, a simple zero-suppression on the spectral calculation can achieve a speed-up of $\mathcal {O}(10^8)$ compared to the time-domain operation. We find empirically that the ratio between the DFT length and the number of significant bins in the spectral kernel, and therefore the achievable speed-up, decreases with $f^{-2}$. At the highest frequencies considered here (5000 Hz), the ratio is still around $10^4$. We note that, due to the volume of data involved, in-memory computation is typically infeasible, thus the speed of I/O operations will have a significant effect for the observed wall-time performances.

Additionally, this number does not take into account the cost of calculating $\tilde{X}_k$ and $\tilde{K}_k^*$. In^22^, the authors suggest to calculate the latter in advance, by performing an FFT on the time-domain kernel for every frequency bin, so that it can be simply applied to the data later. In musical applications, where one usually considers $\mathcal {O}(10^{2-3})$ frequency bins, this will usually result in an effective speed-up because the kernel only needs to be calculated once. Other approaches based on this have been developed, such as re-framing the problem as a convolution and using only small inverse FFTs to retrieve information from every frequency bins^23^ - covering only non-negligible frequency terms. Another approach is to iteratively downsample the data in order to re-use kernel terms and reduce the number of operations at lower frequencies^24^, and yet another application uses neural network implementations to simply perform the transform much faster^25^. However in our case, with a number of frequency bins well exceeding $10^6$, any solution involving repetitions per frequency bin will not end up being advantageous. We however note that iterative downsampling is a possible avenue for future improvements.

The main innovation in this paper is that we instead derive a fully analytical solution for the spectral kernel, rendering the costs of calculating $\tilde{K}_k^*$ negligible. Moreover, using the fact that Eq. (15) applies to any Fourier transform with consistent lengths for both *x* and $\mathcal {K}$, our solution further emulates zero-padding to allow a single $\tilde{X}_k$ to be reused across all frequency bins. More detailed explanations as well as a full derivation can be found in Appendix A.

In summary, using this approach along with our analytical framework allows us to calculate all $X_j$ terms (see Eq. (11)) with a single FFT of the data followed by a heavily zero-suppressed transformation through spectral domain operations. While in theory this constitutes an approximation, because none of the suppressed terms are exactly zero, in practice the difference is negligible and we set a stringent relative threshold of $10^{-5}$ on whether or not to consider values to be zero. Since the kernel is independent of the data, we stress that the (single) FFT is the only pass on the data required in the computation. This approach is thus precise, fast, does not require any post-processing (unlike the approach described in Sec. IIIA), and does not require any kernel pre-computing. Given the data volumes, our implementation also includes an out-of-core FFT algorithm, and small loops are used over large arrays to avoid overloading memory.

Following those improvements, a new computational bottleneck in the PSD calculation emerges: the evaluation of the frequency-dependent normalisation term (see the denominator in Eq. (11)). This is because the (Kaiser) window function we use involves computationally expensive calls to Bessel functions. Closer inspection (see Appendix B) revealed that the whole term is proportional to the segment length ($N_j$, see Eq. (10)). Since this length is already known analytically, this allowed us to pre-compute and re-use it, further reducing costs.

**Table 1:** Commonly referred variables and their values in this analysis.

Variable	Value
*J*	6214612
$f_{min}$	10 Hz
$f_{max}$	5000 Hz
$f_s$	16384 Hz
$\epsilon$	0.01
$\mathcal {Q}$	$10^6$

### ULDM search strategy

**Fig. 3:**
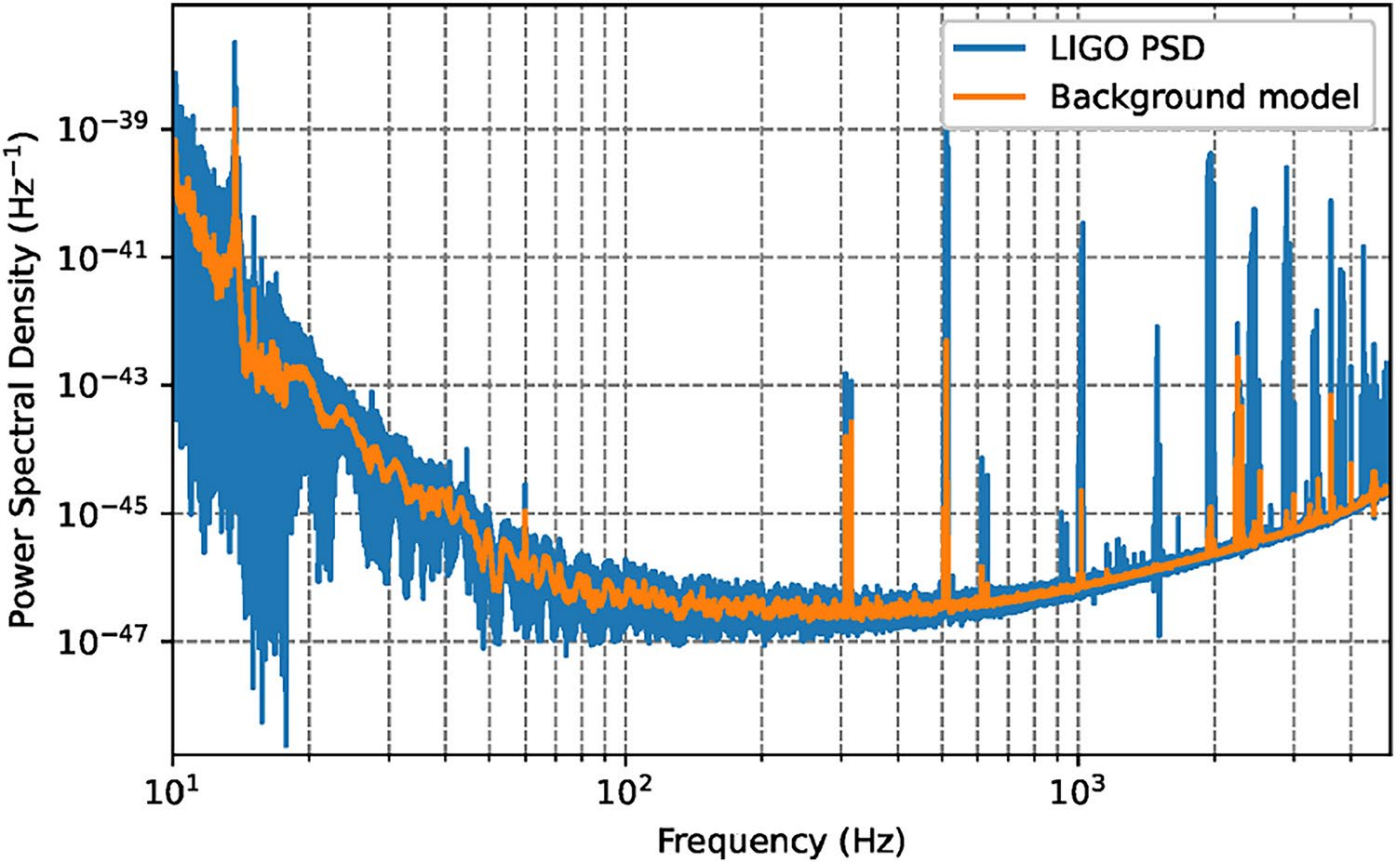
Example LIGO power spectral density (PSD). The overlayed orange line shows the results of the spline fit from the procedure in the text. These fit results, with the variance and skewness of the remaining PSD data, define our background model.

Having calculated PSDs using Eq. (11) and both aforementioned methods, our search for dark matter becomes a search for localized excess in power spectral density. However, since the effect from DM cannot be “turned off”, it is necessary to start by building a background model that is resistant to existing peaks in the data. Similar to methods used by LIGO calibration^38^, we find that a cubic spline in log-log space can well describe the PSD as a function of frequency, see for reference Fig. 3. We further find empirically that the residuals between the log of the PSD and said splines can be well described locally by skew normal (SN) distributions.

**Fig. 4:**
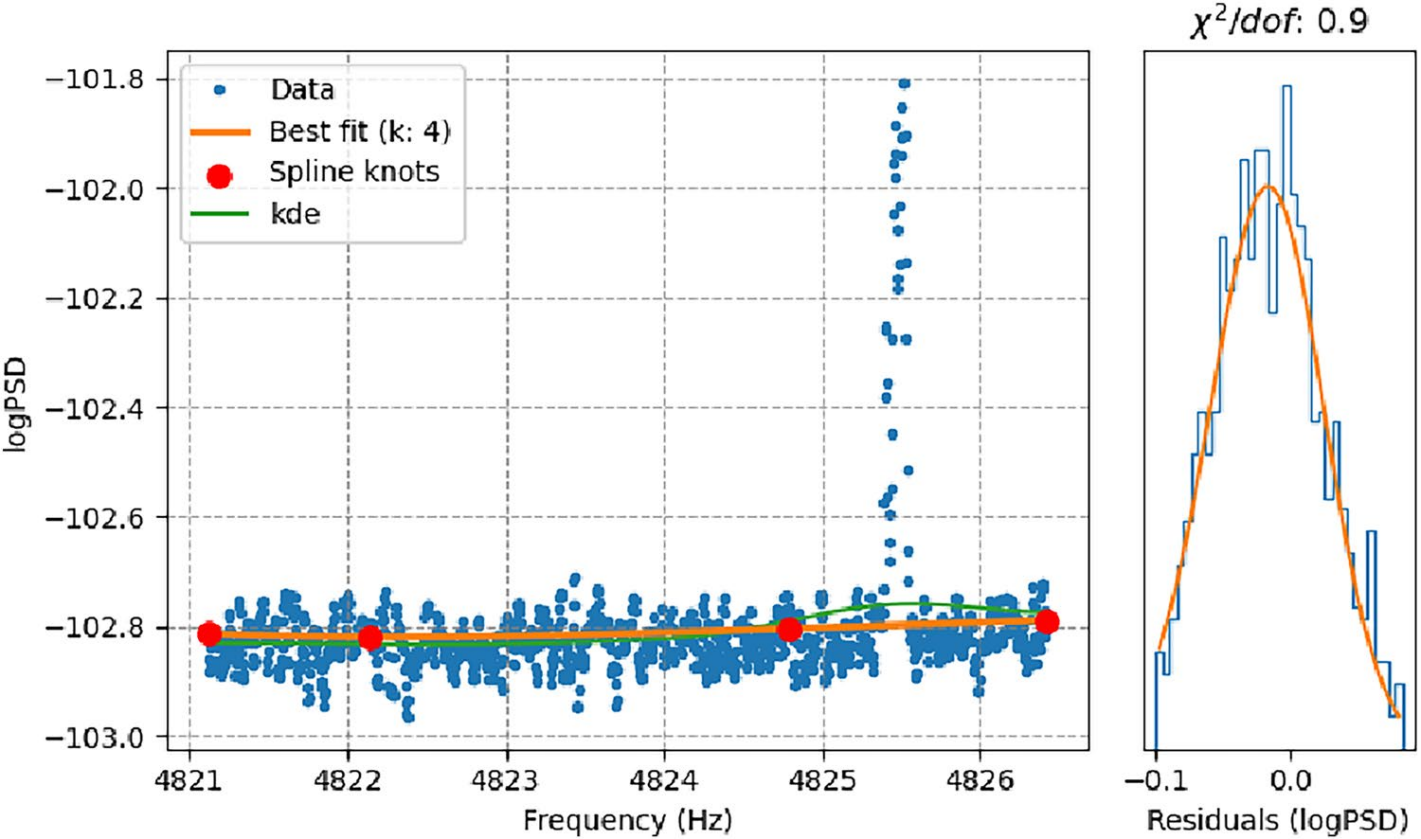
Left) The blue data points represent log(PSD) values as a function of frequency. The narrow green line shows a kernel density estimation (KDE) over these values. The solid orange line indicates the spline fit, with individual knots marked by large red dots. right) distribution of the residuals with accompanying fitted skew normal distribution. As one can see in this example, the combination of spline fits and skew normal distributions can well describe the data while remaining undisturbed by the presence of outliers in the PSD. The difference between KDE and Spline at the lower end of the frequency axis stems from the former not taking the skewness of the distribution into account, and the overestimation on the right side is “leakage” from the peak.

In order to describe the background, one could in theory perform a maximum likelihood fit through an entire PSD. This however encounters a few problems. First is the high number of required variables resulting in an unstable procedure, second is the effect of (known and unknown) so-called lines in the PSDs which would affect naive fitting attempts, and third is the large variation in the properties of LIGO PSDs as a function of frequency. We solve all three issues by implementing a localized procedure in which the PSD is divided into windows of only 1000 frequency bins. Each fit is performed individually within such a window, though window bounds are set to overlap to ensure consistency around boundaries. In each such window, where the SN parameters can safely be viewed as constant, we fit a spline by minimising a skew normal likelihood through the residuals, see Fig. 4. By combining the results from all windows into a single background model, we are able to capture the complexity and frequency-variation of LIGO PSDs. To minimize the effect of existing peaks (presumably including dark matter effects) on the results of those fits, we employ an iterative procedure for each window:

1. Initialize the spline fit with $k = 4$ knots.
2. Minimize the skew normal likelihood.
3. Build a histogram of the residuals and discard all data in bins with heights below 5% of the maximum bin height.
4. Minimize the likelihood again.
5. Compute the Bayesian Information Criterion (BIC).
6. Increase *k* by 1 and repeat steps 2 to 5 until the BIC stops increasing.

This is repeated for each PSD. The removal of low-contributing bins ensures robustness of the fitted background SN parameters against peaks. The Bayesian part of this approach also prevents overfitting, allowing us, as shown in Fig. 4, to reconstruct the overall background shape of the data.

When using the FFT approximation (see Sec. IIIA), an extra step is required. Since the band locations (see Eq. (12)) are known a-priori and because the PSD *drift* (see Fig. 1) scales linearly with frequency, we can simply add lines to the spline fit model. To treat those lines consistently over the frequency axis, one must take care to fit simultaneously over at least an entire band - typically including several windows. Finally, we set a cut on the quality of the fits by using the aforementioned histograms to calculate a $\chi ^2$ value (assuming Poisson uncertainties). We note that this value is not, like the results of a proper $\chi ^2$ fit, statistically expected to fluctuate around the number of degrees of freedom, but we find it is still a good proxy to indicate *goodness-of-fit*. We exclude all data from windows with a corresponding $\chi ^2$ value below 0.5 or above 10.

To combine data from different time segments and interferometers, which would otherwise be challenging due to variations in detector response to DM effects and calibration, we adopt the likelihood-based approach outlined in^4^, where detector responses were modelled using simulated transfer functions. The resulting likelihood is defined as:

16 $$\begin{aligned} \begin{aligned} \log&\mathcal {L}(\mu , \boldsymbol{\theta }, \tilde{\boldsymbol{\theta }}) = \\&\sum _{\text {seg,ifo}} \Bigg [ \sum _{j=0}^{N-1} \log f_{\text {seg,ifo}} \left( g_{\text {seg,ifo}}(\omega _j, \mu , \boldsymbol{\theta }), \tilde{\boldsymbol{\theta }}\right) \\&+ \log \mathcal {N}_{\text {seg,ifo}}\left( \eta _\mathcal {R}(\omega _j)\right) \Bigg ], \end{aligned} \end{aligned}$$

where the subscript *seg* denotes the data segment, *ifo* the interferometer, $\mu = \Lambda _i^{-2}$ is proportional to the amplitude of the DM peak, $\omega _j$ is the frequency in the $j^{th}$ bin, $\eta _\mathcal {R}$ is the ratio between measured and true strain data as determined by LIGO calibration^38^ with a corresponding Gaussian pull term $\mathcal {N}$, and finally $f_{seg,ifo}$ is a skew normal distribution with parameters denoted by $\tilde{\boldsymbol{\theta }}$, with $\boldsymbol{\theta }$ left to describe the spline and calibration parameters. $g_{seg,ifo}$ describes the residuals between the data and the expected background, with terms to model DM and calibration effects:

17 $$\begin{aligned} \begin{aligned} g_{\text {seg,ifo}} =\ &2\log \eta _\mathcal {R} \\&+ \log PSD_\text {seg} \\&- \log \left( y_\text {spline} + \mu \beta _\text {ifo}\right) , \end{aligned} \end{aligned}$$

where $y_{spline}$ refers to the fitted background shape, and $\beta _{ifo}$ is an interferometer-specific calibration term based on^4^. Frequency-dependence throughout the equation is left implicit for simplicity. One should note that this likelihood function assumes that the stochasticity of the DM signal^39,40^ is negligible because, by design, our integration time always matches an entire coherence time.

Within this framework, searching for DM is equivalent to rejecting the hypothesis that $\mu = 0$. We use the profile-likelihood-ratio based test statistic $q_0$ (as described in^41^) which assumes $\mu \ge 0$:

18 $$\begin{aligned} q_0 = {\left\{ \begin{array}{ll} -2 \ln \frac{\mathcal {L}(0, \hat{\hat{\boldsymbol{\theta }}})}{\mathcal {L}(\hat{\mu }, \hat{\boldsymbol{\theta }})} & \text {if } \hat{\mu } \ge 0 \\ 0 & \text {if } \hat{\mu } < 0 \end{array}\right. }, \end{aligned}$$

where $\hat{\hat{\boldsymbol{\theta }}}$ are the values of $\boldsymbol{\theta }$ that maximize the likelihood for a given $\mu$ and ($\hat{\mu }$, $\hat{\theta }$) are the values that globally maximize the likelihood. $q_0$ is zero when $\hat{\mu } < 0$ because since DM cannot induce negative peaks in the PSD, one must not regard under-fluctuations in the data as being incompatible with the null hypothesis.

Conversely, in order to determine upper limits, we adopted the test statistic ${q}_{\mu }$:

19 $$\begin{aligned} q_{\mu } = {\left\{ \begin{array}{ll} -2 \ln \frac{\mathcal {L}(\mu , \hat{\hat{\boldsymbol{\theta }}})}{\mathcal {L}(\hat{\mu }, \hat{\boldsymbol{\theta }})} & \text {if } \hat{\mu } \le \mu \\ 0 & \text {if } \hat{\mu }> \mu \end{array}\right. }, \end{aligned}$$

with the same nomenclature as in Eq. (18). One should note the sign reversal: $q_{\mu }$ is set to zero when $\hat{\mu }> \mu$ because in the case of setting an upper limit, higher values of $\mu$ do not represent incompatibility with the hypothesis. To convert computed values of *q* to significance levels for rejecting the null hypothesis, we first selected a thousand logarithmically spaced frequency bins within our analysis range. We then calculated the significance using both Monte-Carlo sampling of our likelihood and asymptotic formulæ as described in^41^. Finding no statistically significant differences between the two methods, we opted for the latter (faster) one in all subsequent calculations.

## Results and discussion

The data used in this study is identical to that used in^4^, meaning 40 data segments from the third science run of the Hanford and Livingston detectors^42^, selected as those with a continuous measurement length of at least $\tau _c({10}\,\hbox {Hz}) = {27.78}\, \hbox {h}$. This data is calibrated from 10 Hz to 5000 Hz, which is why our results are evaluated in that range. We assume a local DM density of $\rho _{\text {local}} = 0.4\, \hbox {GeV}\, {\hbox {cm}^{-3}}$^43^, and the strategy described in^4^ to find DM candidates on data obtained using both methods described in Sec. III. Finding no viable candidates we construct upper limits (the results, evaluated with the FFT approximation, can be found in^4^). As expected, the spectral *zero-suppression* method shows a consistent improvement ($15\%$ when averaged over all frequencies), as it avoids the approximations required by the *FFT approximation* method. It also avoids the need for additional post-processing which introduces nuisance degrees of freedom to the analysis. After the initial FFT was performed, it is also trivially parallelisable since every frequency bin can be calculated independently. Run-time comparisons between the different methods in our implementation are given in Table 2.

**Table 2:** Cost comparison of estimating a logarithmic PSD on a 28 h LIGO data stretch for different methods. Additional columns show whether the method is parallelisable and whether it provides exact results.

Method	CPU-hr	Parallel	Exact
Brute force	$1.6\cdot 10^5$	$\checkmark$	$\checkmark$
FFT approximation	296	$\times$	$\times$
Zero suppression	123	$\checkmark$	$\checkmark$

We have thus introduced a novel method that performs an efficient constant Q transform without the need for approximations or pre-computation. Additionally, the method is versatile. Indeed, the zero-suppression threshold applied here (see Sec. IIIB) was stringent, and a more relaxed approach could re-introduce some losses while reducing computational costs by at least another order of magnitude. Future work could fine-tune this balance to meet specific targets.

Our method is broadly applicable to any logarithmic spectral calculation, including its original context in computer music, and thus shows promise beyond gravitational-wave physics. This of course includes LIGO dark matter searches^1,2,19,30,44,45^. As mentioned in Sec. I, obtaining optimal results with 200 h of GEO 600 data required well over $10^6$ cpu-hr. This number would have increased by a factor 7 by bringing the lower frequency down to LIGO’s 10 Hz, and by a factor 12 by shifting to LISA’s frequency band. Further, the thousands of hours of available LIGO data, compared to the about 200 h used for GEO600^3^, increase this number by another order of magnitude.

Directly estimating how much our method could improve future experiments’ sensitivities is beyond the scope of this paper. However, it is worth pointing out that while the statistical analyses might look very different (compared to Sec. IIIC). For example, coherence times in the LISA band will be much higher than the mission’s lifetime, and that the analysis could be complicated by e.g. data gaps and a more complex behaviour of the transfer functions. However, ultimately, all future analyses will have to start with a frequency transform that will benefit from our method, which thus has the potential to enhance the scientific yield of next-generation gravitational-wave detectors, whether terrestrial or space-based^21,46–50^.

## Supplementary Information

Supplementary Information.

## Data Availability

The raw calibrated LIGO strain data used to produce our results is readily available at https://gwosc.org/data. Our modified LPSD code, including both methods presented in this paper, is available at github.com/AlexandreGoettel/Scalar-Dark-Matter-LPSD, along with instructions for installation and usage.

## References

[1] Abbott, R. et al. (LVK Collaboration), Constraints on dark photon dark matter using data from LIGO’s and Virgo’s third observing run. *Phys. Rev. D* **105**, 063030. 10.1103/PhysRevD.105.063030 (2022).

[2] Abac, A. G. et al. (LVK Collaboration), Ultralight vector dark matter search using data from the KAGRA O3GK run. *Phys. Rev. D* **110**, 042001. 10.1103/PhysRevD.110.042001 (2024).

[3] Vermeulen, S. M. et al. Direct Limits for Scalar Field Dark Matter from a Gravitational-Wave Detector. *Nature* **600**, 424. 10.1038/s41586-021-04031-y (2021). 34912085 10.1038/s41586-021-04031-y PMC8674151

[4] Göttel, A. S. et al. Searching for Scalar Field Dark Matter with LIGO. *Phys. Rev. Lett.* **133**, 101001. 10.1103/PhysRevLett.133.101001 (2024). 39303225 10.1103/PhysRevLett.133.101001

[5] Grote, H. & Stadnik, Y. V. Novel signatures of dark matter in laser-interferometric gravitational-wave detectors. *Phys. Rev. Res.* **1**, 033187. 10.1103/PhysRevResearch.1.033187 (2019).

[6] Nagano, K., Fujita, T., Michimura, Y. & Obata, I. Axion Dark Matter Search with Interferometric Gravitational Wave Detectors. *Phys. Rev. Lett.* **123**, 111301. 10.1103/PhysRevLett.123.111301 (2019). 31573257 10.1103/PhysRevLett.123.111301

[7] Nagano, K. et al. Axion dark matter search using arm cavity transmitted beams of gravitational wave detectors. *Phys. Rev. D* **104**, 062008. 10.1103/PhysRevD.104.062008 (2021).

[8] Pierce, A., Riles, K. & Zhao, Y. Searching for Dark Photon Dark Matter with Gravitational-Wave Detectors. *Phys. Rev. Lett.* **121**, 061102. 10.1103/PhysRevLett.121.061102 (2018). 30141690 10.1103/PhysRevLett.121.061102

[9] Guo, H. K. et al. Searching for dark photon dark matter in LIGO O1 data. *Commun Phys* **2**, 155. 10.1038/s42005-019-0255-0 (2019).

[10] Michimura, Y., Fujita, T., Morisaki, S., Nakatsuka, H. & Obata, I. Ultralight vector dark matter search with auxiliary length channels of gravitational wave detectors. *Phys. Rev. D* **102**, 102001. 10.1103/PhysRevD.102.102001 (2020).

[11] Nguyen, Q. L., & Miller, A. L. Dark Matter and its Effect on Gravitational Wave Signal. *PoS EPS-HEP2023* 132. 10.22323/1.449.0132 (2024).

[12] Manita, Y., Aoki, K., Fujita, T. & Mukohyama, S. Spin-2 dark matter from an anisotropic universe in bigravity. *Phys. Rev. D* **107**, 104007. 10.1103/PhysRevD.107.104007 (2023).

[13] Lee, C.-H., Primulando, R. & Spinrath, M. Discovery prospects for heavy dark matter in KAGRA. *Phys. Rev. D* **107**, 035029. 10.1103/PhysRevD.107.035029 (2023).

[14] Armaleo, J. M., Nacir, D. L., & Urban, F. R. Searching for spin-2 ULDM with gravitational waves interferometers. *JCAP* **04**, 053.10.1088/1475-7516/2021/04/053.

[15] Derevianko, A. Detecting dark-matter waves with a network of precision-measurement tools. *Phys. Rev. A* **97**, 042506. 10.1103/PhysRevA.97.042506 (2018).

[16] Abbott, R. et al. (LVK Collaboration), Advanced LIGO. *Class. Quantum Grav.* **32**, 074001. 10.1088/0264-9381/32/7/074001 (2015).

[17] Acernese, F. et al. Advanced Virgo: a second-generation interferometric gravitational wave detector. *Class. Quantum Grav.* **32**, 024001. 10.1088/0264-9381/32/2/024001 (2014).

[18] Akutsu, T. et al. Overview of KAGRA: Detector design and construction history. *Prog. Theor. Exp. Phys.* **2021**, 05A101. 10.1093/ptep/ptaa125 (2020).

[19] Miller, A. L. et al. Probing new light gauge bosons with gravitational-wave interferometers using an adapted semicoherent method. *Phys. Rev. D* **103**, 103002. 10.1103/PhysRevD.103.103002 (2021).

[20] Dooley, K. L. et al. (LVK Collaboration), Status of GEO 600. *Journal of Physics: Conference Series* **610**, 012015. 10.1088/1742-6596/610/1/012015 (2015).

[21] Colpi, M. et al. LISA Definition Study Report. arXiv:2402.07571 (2024).

[22] Brown, J. C. & Puckette, M. S. An efficient algorithm for the calculation of a constant Q transform. *J. Acoust. Soc. Am.* **92**, 2698. 10.1121/1.404385 (1992).

[23] Holzmüller, F. et al. Computational efficient realtime capable constant-Q spectrum analyzer. Proceedings of the 148th AES Convention.https://phaidra.kug.ac.at/o:134471 (2020).

[24] Schörkhuber, C., & Klapuri, A. Constant-q transform toolbox for music processing. 7th sound and music computing conference. https://zenodo.org/records/849741 (2010).

[25] Cheuk, K. W. et al. nnAudio: An on-the-fly GPU Audio to Spectrogram Conversion Toolbox Using 1D Convolution Neural Networks. arXiv:1912.12055 (2020).

[26] Arvanitaki, A., Huang, J. & Van Tilburg, K. Searching for dilaton dark matter with atomic clocks. *Phys. Rev. D* **91**, 015015. 10.1103/PhysRevD.91.015015 (2015).

[27] Stadnik, Y. V. & Flambaum, V. V. Can Dark Matter Induce Cosmological Evolution of the Fundamental Constants of Nature?. *Phys. Rev. Lett.* **115**, 201301. 10.1103/PhysRevLett.115.201301 (2015). 26613429 10.1103/PhysRevLett.115.201301

[28] Ringwald, A. Exploring the role of axions and other WISPs in the dark universe. *Dark Universe* **1**, 116. 10.1016/j.dark.2012.10.008 (2012).

[29] Hees, A. et al. Violation of the equivalence principle from light scalar dark matter. *Phys. Rev. D* **98**, 064051. 10.1103/PhysRevD.98.064051 (2018).

[30] Morisaki, S. et al. Improved sensitivity of interferometric gravitational-wave detectors to ultralight vector dark matter from the finite light-traveling time. *Phys. Rev. D* **103**, L051702. 10.1103/PhysRevD.103.L051702 (2021).

[31] Manita, Y. et al. Exploring the spin of ultralight dark matter with gravitational wave detectors. *Phys. Rev. D* **109**, 095012. 10.1103/PhysRevD.109.095012 (2024).

[32] Catena, R. & Ullio, P. The local dark matter phase-space density and impact on WIMP direct detection. *JCAP* **05**, 005. 10.1088/1475-7516/2012/05/005 (2012).

[33] Cautun, M. et al. The milky way total mass profile as inferred from Gaia DR2. *MNRAS* **494**, 4291. 10.1093/mnras/staa1017 (2020).

[34] Deason, A. J. et al. The local high-velocity tail and the Galactic escape speed. *MNRAS* **485**, 3514. 10.1093/mnras/stz623 (2019).

[35] Tröbs, M. & Heinzel, G. An algorithm for the machine calculation of complex Fourier serie. *Measurement* **39**, 120. 10.1016/j.measurement.2005.10.010 (2006).

[36] Welch, P. The use of fast Fourier transform for the estimation of power spectra: A method based on time averaging over short, modified periodograms. *IEEE Trans. Audio Electroacoustics* **15**, 70. 10.1109/TAU.1967.1161901 (1967).

[37] Piccinni, O. J. et al. A new data analysis framework for the search of continuous gravitational wave signals. *Class. Quantum Grav.* **36**, 015008. 10.1088/1361-6382/aaefb5 (2018).

[38] Sun, L. et al. Characterization of systematic error in Advanced LIGO calibration. *Class. Quantum Grav.* **37**, 225008. 10.1088/1361-6382/abb14e (2020).

[39] Nakatsuka, H. Stochastic effects on observation of ultralight bosonic dark matter. et al. *Phys. Rev. D* **108**, 092010.10.1103/PhysRevD.108.092010 (2023).

[40] Centers, G. P. Stochastic fluctuations of bosonic dark matter. et al., *Nat. Commun.* **12**, 7321. 10.1038/s41467-021-27632-7 (2021). 10.1038/s41467-021-27632-7 PMC8677790 34916510

[41] Cowan, G. et al. Asymptotic formulae for likelihood-based tests of new physics. *Eur. Phys. J. C* **71**, 1554. 10.1140/epjc/s10052-011-1554-0 (2011).

[42] Abbott, R. et al. (LVK Collaboration), Open Data from the Third Observing Run of LIGO, Virgo, KAGRA, and GEO. *Astrophys. J. Suppl.* **267**, 29. 10.3847/1538-4365/acdc9f (2023).

[43] Read, J. I. The local dark matter density. *J. Phys. G: Nucl. Part. Phys.* **41**, 063101. 10.1088/0954-3899/41/6/063101 (2014).

[44] Miller, A. L. & Mendes, L. First search for ultralight dark matter with a space-based gravitational-wave antenna: LISA Pathfinder. *Phys. Rev. D* **107**, 063015. 10.1103/PhysRevD.107.063015 (2023).

[45] Miller, A. L., Badaracco, F. & Palomba, C. Distinguishing between dark-matter interactions with gravitational-wave detectors. *Phys. Rev. D* **105**, 103035. 10.1103/PhysRevD.105.103035 (2022).

[46] Abac, A. et al. The Science of the Einstein Telescope. arXiv:2503.12263 (2025).

[47] Reitze, D. et al. Cosmic Explorer: The U.S. Contribution to Gravitational-Wave Astronomy beyond LIGO. *Bull. Am. Astron. Soc.* **51**, 7-035. 10.48550/arXiv.1907.04833 (2019).

[48] Hu, W.-R. & Wu, Y.-L. The Taiji Program in Space for gravitational wave physics and the nature of gravity. *Nat. Sci. Rev.* **4**, 685. 10.1093/nsr/nwx116 (2017).

[49] Luo, J. et al. TianQin: a space-borne gravitational wave detector. *Class. Quantum Grav.* **33**, 035010. 10.1088/0264-9381/33/3/035010 (2016).

[50] Nuttall, A. Some windows with very good sidelobe behavior. *IEEE Trans. Acoust. Speech Signal Process.* **29**, 84. 10.1109/TASSP.1981.1163506 (1981).

