# Supplementary material for: Analytical kernels for efficient constant Q transforms in dark matter searches with LIGO
Source: Sci Rep. 2026 Apr 1;16:15364. doi: 10.1038/s41598-025-33428-2 (PMC13183897; doi:10.1038/s41598-025-33428-2)
Supplement: Supplementary file 1 — Supplementary Information. [file 41598_2025_33428_MOESM1_ESM.pdf]

SUPPLEMENTAL MATERIAL

Appendix A

The time kernel we wish to analytically Fourier transform is the product of our window function and an exponential term, as defined in Eq. (14). In this study we use the Kaiser-Bessel window [51], defined as:

$$w(x) = \frac{1}{LI_0(\beta)} I_0\left(\beta\sqrt{1-(2x/L)^2}\right) \text{ for } |x| < L/2, \tag{A1}$$

where the window is of length L and β is a scaling parameter that allows some level of control over main and side lobe levels. This window was designed to be an analytical close-to-optimum solution to maximise total power in the main lobe. We use $\beta = 30$. The window and the value of β were chosen for compatibility with [3, 4]. The Fourier transform of the Kaiser-Bessel window is given by:

$$\mathcal{F}(w)(f) = \begin{cases} \frac{\sin\sqrt{(\pi Lf)^2-\beta^2}}{I_0(\beta)\sqrt{(\pi Lf)^2-\beta^2}}, & (\pi Lf)^2 - \beta^2 \geq 0 \\ \frac{\sinh\sqrt{\beta^2-(\pi Lf)^2}}{I_0(\beta)\sqrt{\beta^2-(\pi Lf)^2}}, & \text{otherwise,} \end{cases} \tag{A2}$$

where f is the frequency. The two cases above are mathematically equivalent but shown separately to reflect software implementation. This solution lets us easily compute the spectral kernels because the aforementioned exponential term can simply be interpreted as a discrete shift in frequency space. However, in order to use these results, because Eq. (A2) only contains real terms, we must ensure that the spectral kernel is real, *i.e.* that the time kernel is conjugate symmetric. We achieve this by following the approach in [22], implementing a discrete version of the window that is symmetric around the centre of the interval. Assuming an odd window length N this can be written as:

$$\begin{aligned} w'(n) &= w(n-(N-1)/2), \\ &= \begin{cases} \frac{I_0\left(\beta\sqrt{1-\left(\frac{2n}{N-1}-1\right)^2}\right)}{I_0(\beta)} & \text{if } 0 \leq n \leq N \\ 0 & \text{otherwise.} \end{cases} \end{aligned} \tag{A3}$$

Applying the same transformation $n \to n-(N-1)/2$ to the exponential term of the time kernel results in a conjugate symmetric time kernel, allowing us to directly evaluate our spectral kernel.

However, it also induces the need for an *a posteriori* correction to align the time domain data as required by our PSD calculation in Eq. (6). This can be understood with the sketch below:

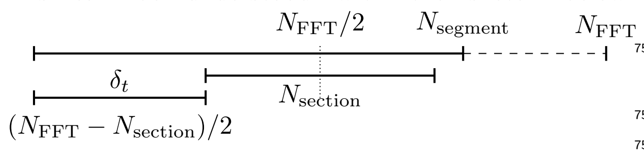

where (NOT to scale) $N_{\rm segment}$ is the number of data points in a given strain segment, $N_{\rm FFT}$ is the lowest power of two that is equal or higher than $N_{\rm segment}$, and $N_{\rm section}$ is the frequency-dependent length of the sections being summed over (see III B). The kernel (see Eq. (A3)) now defined as symmetric around the centre of the interval, which is in practice defined by $N_{\rm FFT}$, would describe the data under the line labelled $N_{\rm section}$. It is possible to align the window to the beginning of our data segment by shifting it by $\delta_t = (N_{\rm FFT} - N_{\rm section})/2$. We achieve this by multiplying the previously-obtained spectral kernel by a corresponding $e^{2\pi in\delta_t}$ term. By shifting the time kernel through this retroactive multiplication of the spectral kernel, we can keep its necessary complex-conjugate properties as derived above during the Fourier transform step, but are still able to implement the sum over sections required by Eq. (11).
Considering all the above and after simplification, the spectral kernel becomes:

$$\tilde{K}_j = \frac{1}{I_0(\beta)} \frac{\sin\sqrt{\left[\pi(N_j-1)\left(\frac{j}{N_{\rm FFT}} - \frac{\mathcal{Q}}{N_j}\right)\right]^2 - \beta^2}}{\sqrt{\left[\pi(N_j-1)\left(\frac{j}{N_{\rm FFT}} - \frac{\mathcal{Q}}{N_j}\right)\right]^2 - \beta^2}}, \tag{A4}$$

where $(N_j - 1)$ terms arise from the coordinate transform, $j/N_{\rm FFT}$ stems from finding the equivalent frequency to $\tilde{X}_j$ after its FFT (with $f_{{\rm FFT},j} = jf_s/N_{\rm FFT}$), and the $\mathcal{Q}/N_j$ terms remains after the discrete frequency-space shift. Inserting all of the previous steps in Eq. (14), the power terms can thus be expressed analytically as:

$$X_{j,s} = \frac{1}{N}\sum_{j=0}^{N_j-1} \tilde{X}_j \cdot \tilde{K}_{j'} \cdot e^{\frac{2\pi ij}{N_{\rm FFT}} N'(j,s)},$$

where:

$$\begin{aligned} j' &= j/N_{\rm FFT} - \mathcal{Q}/N_j \\ N'(j,s) &= \frac{N_{\rm FFT} - N_j}{2} - s\delta_s(j), \end{aligned} \tag{A5}$$

where the index $s \in [0, ..., N_{\rm sections}]$ was added to show how we sum over data sections in the Welch averaging, and with $\delta_s(j) = (1-\xi) \cdot N_j$ as the distance between data sections for a given overlap ξ. The $j/N_{\rm FFT} - \mathcal{Q}/N_j$ term stems from interpreting the exponential component in the time kernel as a discrete shift in frequency space, the $(N_{\rm FFT} - N_j)/2$ term makes use of the same discrete shift, but in time space, to ensure that the summation over the windows correctly starts at index 0. Finally, the $s\delta_s(j)$ term is used to shift the data over the different sections to perform the averaging as detailed in Eq. (11).

Appendix B

The calculation of the total power in a given frequency bin, see Eq. (11), requires a normalisation term [35] in

which a full window function must be evaluated in each frequency bin. Since we are using a Kaiser-Bessel window function, each of those terms requires a call to the modified Bessel function of zeroth order. This is a slow operation, and while its cost was bearable in past analyses, given the speed-up factors achieved in this study, it becomes a bottleneck. We thus strive to calculate this term analytically and begin by setting:

$$\sum_{n=0}^{N_j-1} w_n^2 \approx \frac{1}{I_0^2(\beta)} \int_0^{N_j} dn\, I_0^2\left(\beta\sqrt{1-\left(\frac{2n}{N_j-1}-1\right)^2}\right). \tag{B1}$$

This is a very natural approximation given the integration lengths (10^{5-9}) in our analysis. We can then use the summation notation of the Bessel function to take the Cauchy product of I_0^2:

$$\begin{aligned} I_0^2(x) &= \sum_{k=0}^{\infty}\sum_{l=0}^{k} \frac{1}{(l!(k-l)!)^2} \cdot \left(\frac{x}{2}\right)^{2k} \\ &= \frac{1}{\sqrt{\pi}} \sum_{k=0}^{\infty} \frac{x^{2k}}{(k!)^3} \Gamma\left(\frac{2k+1}{2}\right). \end{aligned} \tag{B2}$$

Implementing this result into Eq. (B1) and shifting the integral inside of the sum, we continue with:

$$\begin{aligned} &= \frac{1}{\sqrt{\pi}} \sum_{k=0}^{\infty} \frac{\Gamma\left(\frac{2k+1}{2}\right)}{(k!)^3} \int_0^{N_j} dn\, \beta^{2k} \left(1-\left(\frac{2n}{N_j}-1\right)^2\right)^k \\ &= N_j \frac{1}{\sqrt{\pi}} \sum_{k=0}^{\infty} \frac{\Gamma\left(\frac{2k+1}{2}\right)}{(k!)^3} (2\beta)^{2k} \mathcal{B}(k+1, k+1), \end{aligned} \tag{B3}$$

where $\mathcal{B}$ is the beta function, and we have omitted the $I_0^2(\beta)$ term for simplicity. While this can be evaluated numerically, we find that the relevant result here is that for a fixed β, the sum becomes fully proportional to N_j. In this analysis, we can thus calculate the full normalisation for a single frequency bin, and then simply scale it to all other values of N_j.
